# Serum N-glycomic profiling may provide potential signatures for surveillance of COVID-19

**DOI:** 10.1093/glycob/cwac051

**Published:** 2022-08-04

**Authors:** Yongjing Xie, Michael Butler

**Affiliations:** National Institute for Bioprocessing Research and Training, Foster Avenue, Mount Merrion, Blackrock, Co., Dublin, A94 X099, Ireland; National Institute for Bioprocessing Research and Training, Foster Avenue, Mount Merrion, Blackrock, Co., Dublin, A94 X099, Ireland

**Keywords:** COVID-19, InstantPC, protein glycosylation, SARS-CoV-2, serum glycomics

## Abstract

Disease development and progression are often associated with aberrant glycosylation, indicating that changes in biological fluid glycome may potentially serve as disease signatures. The corona virus disease-2019 (COVID-19) pandemic caused by severe acute respiratory syndrome coronavirus 2 (SARS-CoV-2) represents a significant threat to global human health. However, the effect of SARS-CoV-2 infection on the overall serum N-glycomic profile has been largely unexplored. Here, we extended our 96-well-plate-based high-throughput, high-sensitivity N-glycan profiling platform further with the aim of elucidating potential COVID-19-associated serum N-glycomic alterations. Use of this platform revealed both similarities and differences between the serum N-glycomic fingerprints of COVID-19 positive and control cohorts. Although there were no specific glycan peaks exclusively present or absent in COVID-19 positive cohort, this cohort showed significantly higher levels of glycans and variability. On the contrary, the overall N-glycomic profiles for healthy controls were well-contained within a narrow range. From the serum glycomic analysis, we were able to deduce changes in different glycan subclasses sharing certain structural features. Of significance was the hyperbranched and hypersialylated glycans and their derived glycan subclass traits. T-distributed stochastic neighbor embedding and hierarchical heatmap clustering analysis were performed to identify 13 serum glycomic variables that potentially distinguished the COVID-19 positive from healthy controls. Such serum N-glycomic changes described herein may indicate or correlate to the changes in serum glycoproteins upon COVID-19 infection. Furthermore, mapping the serum N-glycome following SARS-CoV-2 infection may help us better understand the disease and enable “Long-COVID” surveillance to capture the full spectrum of persistent symptoms.

## Introduction

The corona virus disease-2019 (COVID-19) pandemic caused by severe acute respiratory syndrome coronavirus 2 (SARS-CoV-2) (Huang et al. 2020; Yang et al. 2020) has represented the most significant threat to global human health. As of 1 June 2022, more than 530 million confirmed cases with more than 6.29 million death were recorded according to data from John Hopkins University (https://coronavirus.jhu.edu/map.html) (Dong et al. 2020). Knowledge about SARS-CoV-2 has increased substantially since it was first reported, including its emergence, genome and structure, detection and characterization, transmission, receptor recognition, cell entry, replication, pathogenesis, vaccine development, recent therapeutic strategies, and even post-acute sequelae (termed as “Long-COVID”) (Doykov et al. 2020; Harrison et al. 2020; Seyed Hosseini et al. 2020; Shang et al. 2020; Al-Aly et al. 2021; Boopathi et al. 2021; Mandal et al. 2021; V'Kovski et al. 2021).

As one of the most abundant and structurally diverse post-translational modifications, protein glycosylation is involved in numerous cellular mechanisms that contribute to health and disease, and has been found to regulate protein folding, cell recognition and adhesion, molecular trafficking and clearance, receptor activation, signal transduction and endocytosis (Ohtsubo and Marth 2006; Reily et al. 2019). Therefore, glycosylation is highly sensitive to the pathological environment and has been implicated in various diseases, such as cancer, genetic diseases, autoimmunity, and chronic inflammation (Freeze 2013, Wiederschain 2022). More relevant to virus infection, protein glycosylation has been recognized to play a critical role in facilitating virus evasion from the innate and adaptive immune responses (Casalino et al. 2020; Grant et al. 2020; Zhao et al. 2021). It has been well known that the glycosylation of viral envelop proteins is essential for infectivity and affects immune recognition (Vigerust and Shepherd 2007; Bagdonaite and Wandall 2018; Watanabe et al. 2019; Li et al. 2021). Therefore, glycomics has gradually gained extensive interest in biomedical research and drug discovery (Turnbull and Field 2007; Hart and Copeland 2010). Additionally, both the trimeric spike protein (S) of SARS-CoV-2 and its human angiotensin converting enzyme 2 (hACE2) receptor are heavily glycosylated, including at sites near their binding interface (Lan et al. 2020; Watanabe et al. 2020; Yan et al. 2020; Shajahan et al. 2021). Binding of the spike protein to the hACE2 receptor triggers the translocation of the virus into host cells (Letko et al. 2020). Consequently, serum-based glycomic profiles may well be altered following SARS-CoV-2 infection. Such glycomic profiles have often been valuable as signatures of various diseases and may enable a better understanding of how vaccination affects immunogen processing and presentation, and eventually for therapeutic strategies development (An et al. 2009, Lebrilla and An 2009, Varki 2022). However, the effect of SARS-CoV-2 infection on the overall serum glycomic profiles has been largely unexplored.

The structural complexity of glycans has hampered the analysis of glycomic profiles, as the conventional hydrophilic interaction liquid chromatography coupled with fluorescence detection (HILIC-FLD) method after 2-aminobenzamide (2-AB) derivatization are labor-intensive, time-consuming, with poor analytical resolution and reproducibility (Li 2010; Everest-Dass et al. 2018). Therefore, the development of high-throughput and high-sensitivity technologies for reliable analysis of human serum glycomic profile can be a valuable tool in the study of disease or viral infection. In the current study, we extended our previously developed 96-well-plate-based high-throughput, high-sensitivity glycan preparation platform (Xie et al. 2021), and related serum N-glycan identification and analytical method (Xie and Butler 2022) even further to COVID-19 research with the aim of elucidating any potential COVID-19-associated serum N-glycomic changes and consequently gain better understand of this disease. This N-glycan preparation and analysis platform allows highly accurate serum N-glycomic profiling with minimum sample preparation. The entire preparation for up to 96 serum samples (1 μL of serum) per plate takes a maximum of only 1 h to completion. The method is sufficiently sensitive to profile up to 100 N-glycan structures, 46 major glycan peaks (GPs), and 16 glycan subclass traits in each serum sample.

Using this platform, the serum N-glycomic profiles from a cohort of COVID-19 positive (23) were analyzed and compared with those from healthy individuals (10). It was observed that the total glycan quantity from the COVID-19 positive cohort was significantly higher than that in the healthy controls, with particular enhancement of hyperbranched and hypersialylated glycans and subclass traits. Additionally, the serum N-glycomic map revealed the substantial downregulation of 3 N-GPs and up-regulation of 8 GPs in the COVID-19 positive cohort. Although the investigated sample size was relatively small, these statistically significant differences indicate the potential of serum N-glycomic mapping described herein to be used as a supplementary technique for surveillance of SARS-CoV-2 infection. Eventually, this may help us better understand the disease and enable surveillance of persistent symptoms that are often referred to as “Long-COVID.”

## Results

### High-throughput and high-sensitivity platform for functional serum N-glycomic profiling

In the current study, we extended our previously described high-throughput, high-sensitivity N-glycan preparation platform (Xie et al. 2021) further to identify potential alterations in human serum N-glycome upon or after infection with SARS-CoV-2. Table 1 lists the 23 COVID-19 positive serum samples, 10 healthy serum samples, and the source information that was supplied regarding the sex and age of the serum donors. We analyzed each sample for sub-type antibodies (IgG, IgM, and IgA) against SARS-CoV-2 spike protein subunit 1 (S1) receptor-binding domain (RBD) protein using an indirect enzyme-linked immunosorbent assay (Ollis et al. 2015) protocol described in the Materials and Methods section. Positive values were obtained for all 23 COVID-19 serum samples using anti-SARS-CoV-2 S1 RBD protein IgG, IgM, and IgA as standards, and the mean positive values (units/mL) were 129.05 (Ke et al. 2020), 370.46 (IgM), and 97.59 (Zhu et al. 2020). It is to be noted that these units are relative to the individual anti-SARS-CoV-2 S1 RBD protein IgG, IgM, and IgA standards provided by the supplier (Ray Biotech, Peachtree Corners, Georgia, USA), but the absolute quantities of each are unknown. No anti-SARS-CoV-2 S1 RBD protein was detected in IgG, IgM, or IgA of the healthy serum samples.

**Table 1:** Representative clinical information for COVID-19 positive and negative serum samples.

Sample ID	Patient ID	Sex	Age	COVID-19 test result	IgG S1RBD (unit/mL)	IgM S1RBD (unit/mL)	IgA S1RBD (unit/mL)
P1	PS302	F	67	Positive	48.71	540.30	5.72
P2	PS303	M	76	Positive	28.21	329.69	103.86
P3	PS304	M	85	Positive	121.13	233.52	346.43
P4	PS305	F	76	Positive	254.84	670.03	81.59
P5	PS308	M	50	Positive	188.81	259.83	114.99
P6	PS310	F	76	Positive	232.80	564.57	8.28
P7	PS313	F	69	Positive	209.44	63.43	16.15
P8	PS314	F	64	Positive	92.04	149.85	27.39
P9	PS326	M	58	Positive	7.95	197.39	24.78
P10	PS333	M	54	Positive	16.09	765.44	45.39
P11	PS346	F	80	Positive	174.37	80.29	147.46
P12	PS347	F	83	Positive	14.46	802.96	9.77
P13	PS348	F	76	Positive	11.52	0.00	2.95
P14	PS351	M	67	Positive	11.95	84.08	21.57
P15	PS357	F	74	Positive	0.59	49.59	159.52
P16	PS358	F	63	Positive	1.20	155.57	141.25
P17	PS359	M	61	Positive	18.49	1322.20	201.36
P18	PS602	F	30	Positive	51.37	74.30	17.16
P19	PS603	F	21	Positive	30.40	52.19	16.57
P20	PS616	F	59	Positive	189.90	479.01	123.78
P21	PS327	M	89	Positive	549.43	614.44	163.61
P22	PS329	F	92	Positive	692.30	426.76	267.82
P23	PS377	M	75	Positive	22.25	605.03	197.26
H1	SN204	M	46	Negative	–	–	–
H2	SN205	F	22	Negative	–	–	–
H3	SN206	M	18	Negative	–	–	–
H4	SN207	M	47	Negative	–	–	–
H5	SN208	F	25	Negative	–	–	–
H6	SN209	M	65	Negative	–	–	–
H7	SN210	M	28	Negative	–	–	–
H8	SN211	M	63	Negative	–	–	–
H9	SN212	F	38.7	Negative	–	–	–
H10	SN213	M	36.5	Negative	–	–	–

M: Male, F: Female, “–”: No values or not detected. COVID-19 Positive: Serum samples from COVID-19 patients confirmed by RT-PCR, antigen, and/or antibody serology tests. COVID-19 Positive: Serum samples from COVID-19 patients confirmed by RT-PCR, antigen, and/or antibody serology tests. COVID-19 Negative: serum samples from patients who do not have COVID-19. IgG S1RBD: IgG antibody to the SARS-CoV-2 spike S1 RBD protein in human serum. IgM S1RBD: IgM antibody to the SARS-CoV-2 spike S1 RBD protein in human serum. IgA S1RBD: IgA antibody to the SARS-CoV-2 spike S1 RBD protein in human serum.

**Fig. 1:**
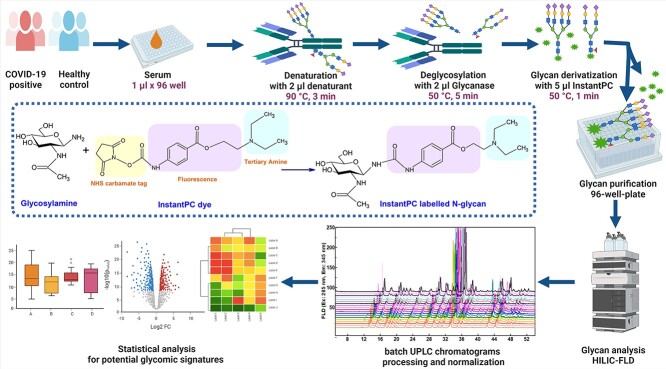
Workflow for high-throughput and high-sensitivity human serum N-glycosylation profiling for untargeted screening of glycomic features for COVID-19 surveillance. Human serum (1 μL) from COVID-19 positive patients and healthy controls were processed and then analyzed by HILIC-FLD, followed by chromatograms batch processing and normalization and statistical analysis for identification of potential glycomic signatures. The activated carbamate chemistry based reaction scheme for InstantPC labelling glycosylamine is displayed. Created by ACD/ChemSketch and BioRender.com.

**Fig. 2:**
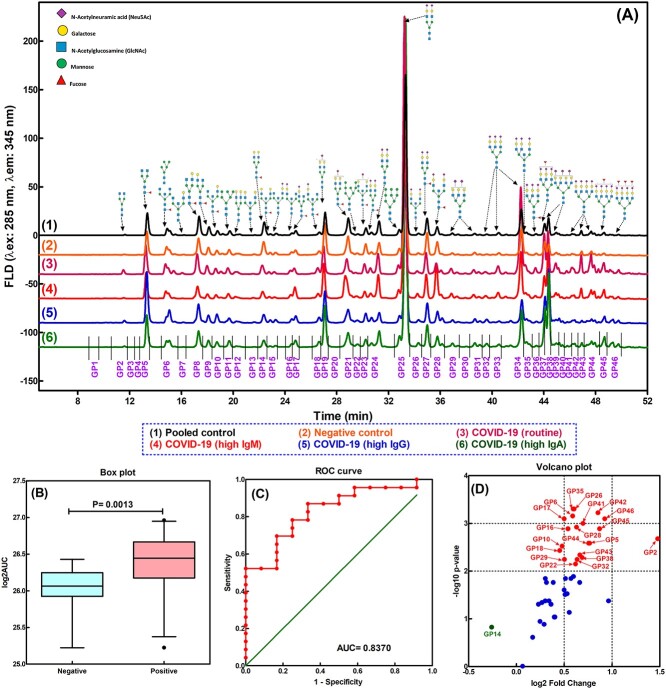
Direct comparison of human serum N-glycome between COVID-19 positive patients and healthy controls. A) Representative HILIC-FLD chromatograms of human serum N-glycome labelled with InstantPC for selective COVID-19 positive patients with high IgA, IgG, IgM content, and healthy controls, respectively. B) Box plot of log2 transformed AUC for total 46 GPs (log2AUC). *Y*-axis represents log2 transformed AUC. The lower and upper bars connected to each box indicate the boundaries of the normal distribution and the lower and upper box edges mark the first and third quartile boundaries within each distribution. The bold line within the box indicates the median value of the distribution. Outliers are labelled as black-filled circle. C) ROC curve of the log2 transformed AUC of the 46 GPs with the AUC for the ROC curve as 0.8370, and *P* = 0.0015. D) Volcano plot (*X*-axis represents the log2 of the fold change, *Y*-axis represents the negative decade logarithm of the significance value *P*) for the quantified GPs indicating significant changes of log2AUC for individual GPs. Two *P*-value thresholds are indicated (*P* = 0.01 and 0.001). The significant glycan variables above the first threshold were considered as significantly changed and labelled in red. The down-regulated GP was labelled in green. The rest of the GPs with *P* > 0.01 were considered as not significantly changed and labelled in blue.

As shown in Figure 1, to facilitate potential application for point of care (POC) testing ideally by using finger pricking blood, the volume of human serum has been reduced significantly to only 1 μL. And it has been cross-validated to confirm that this workflow was robust, reliable, with good reproducibility. The coefficient of variation (CV) of the integrated area under the curve (AUC) generated from triplicate serum samples for total GPs after HILIC-FLD analysis was calculated to be only 0.0133. Therefore, it is reasonable to deduce that any significant changes in the HILIC-FLD chromatograms are due to the human serum under investigation rather than to error of sampling or analytical artifact. Additionally, the 2 cohort samples (healthy and COVID-19 infected) demonstrated similar comparable N-glycan profiles under the chosen chromatographic conditions, with a total of 46 well-resolved GP identified (Fig. 2A). This kind of N-glycomic profile is typical of human serum regardless of disease status, sex, age, or body mass index (BMI), with GP25 (assigned as A2G2S2) as the dominant GP and GPs 5, 8, 14, 19, 21, 24, 27, 28, 34, 37, and 38 as the relatively more abundant GPs.

### Overall serum N-glycome elevated significantly in COVID-19 positive cohort

Although the serum N-glycome from both cohorts demonstrated similar and comparable GPs, the overall integrated peak areas (or heights) from the COVID-19 positive cohort (selective chromatograms 3, 4, 5, and 6 in Fig. 2A) were significantly greater (or higher) than those from both the blank pooled serum control and individual healthy controls where the N-glycan profiles were almost identical (selective chromatograms 1 and 2 in Fig. 2A). Since the InstantPC fluorescent label binds with each glycosylamine intermediate at an one-to-one (1:1) molar ratio (as shown in the reaction scheme in Fig. 1), the quantification of the N-glycans can be made from the measurement of the integrated area under each peak (AUC). This allowed us to carry out a direct comparison between these 2 groups. As shown in the box plot in Figure 2B, the log2 transformed total AUC (log2AUC) for all the 46 identified GPs from the COVID-19 positive group was substantially higher than that from the healthy control group, with the median values as 26.568 and 26.063, respectively. Additionally, the log2AUC values were further analyzed by the receiver operating characteristic (Behnke et al. 2021) test and Mann–Whitney test to evaluate the ability to distinguish COVID-19 positive patients from healthy controls based on the generated AUC and *P* values. It has become clear that log2AUC held great potential to differentiate the 2 cohorts (AUC for ROC curve = 0.8370, *P* = 0.0013, as shown in Fig. 2B and C).

### COVID-19 positive cohort demonstrated substantial serum N-glycomic variability

**Fig. 3:**
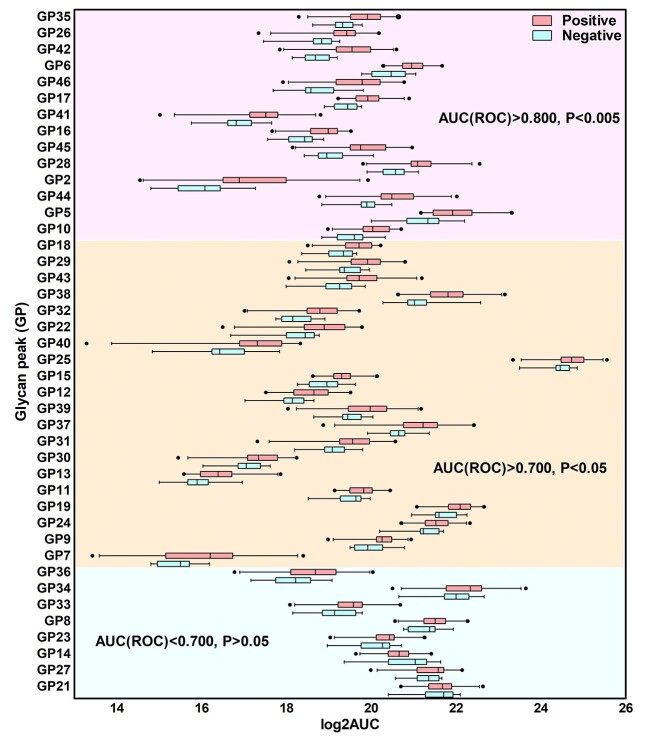
Direct comparison of individual human serum GP abundance between COVID-19 positive patients and healthy controls. *X*-axis represents log2 transformed AUC for each GP, and *Y*-axis represents each GP. The left and right bars connected to each box indicate the boundaries of the normal distribution and the left and right box edges mark the first and third quartile boundaries within each distribution. The bold line within the box indicates the median value of the distribution. Outliers are labelled as black filled circle. The GPs demonstrating excellent (with AUC for ROC analysis > 0.80, and *P* value for Mann–Whitney test < 0.005), acceptable (AUC > 0.70, *P* < 0.05), and no (AUC < 0.70, *P* > 0.05) diagnostic performance accuracy was displayed in top (shaded in pink), middle (shaded in light yellow), and bottom (shaded in light cyan) panel, respectively.

We further analyzed the serum N-glycomic profiles to see if single or multiple GPs were significantly altered in the COVID-19 positive group when compared with the healthy control group. No specific GPs belonged exclusively to either the COVID-19 positive or the healthy control group. However, the log2AUC values of the GPs from the COVID-19 positive cohort demonstrated significant difference from those of the healthy controls. As shown in Fig. 2D the volcano plot, except GP14 (assigned as FA2G2) where it was identified as down-regulated, majority of the GPs were up-regulated in COVID-19 positive patients when compared with healthy controls. GPs 6, 17, 26, 35, 41, 42, and 46 were elevated significantly (*P* < 0.001, −log10 *P*-value > 3), while GPs 2, 5, 10, 16, 18, 22, 28, 29, 32, 38, 43, 44, and 45 were increased to a lesser extent (*P* < 0.01, −log10 *P*-value > 2). This was further supported by Figure 3 box plot and Supplementary Information ST IV, where GPs 35, 26, 42, 6, 46, 17, 41, 16, 45, 28, 2, 44, 5, and 10 demonstrated excellent diagnostic performance to distinguish the COVID-19 positive cohort from healthy controls (AUC for ROC curve > 0.80, *P* < 0.005). GPs 18, 29, 43, 38, 32, 22, 40, 25, 15, 12, 39, 37, 31, 30, 13, 11, 19, 24, 9, and 7 also demonstrated an acceptable diagnostic performance (AUC for ROC curve > 0.70, *P* < 0.05). Reference to the glycan assignment as shown in Figure 2A and Supplementary Information ST II, most of the up-regulated GPs were assigned as di-, tri-, and tetra-sialylated glycans, except GPs 2, 5, 6, and 10 that were neutral glycans. The rest of the GPs were up-regulated but did not demonstrate an acceptable diagnostic performance (AUC for ROC curve < 0.70, *P* > 0.05).

The interquartile range (IQR) is usually used as an indicator for variability of a dataset (Zwillinger and Kokoska 2000; Ross 2010). As shown in Supplementary Information ST IV, the IQR for majority of the log2(GP)s (including the glycan subclasses and relative abundance to be discussed below) from the COVID-19 positive cohort was noticeably wider compared to the healthy counterparts. For example, the IQR for log2GP44 was 0.8415 for COVID-19 positive cohort, while its value was only 0.2736 for the healthy control group. Similarly, the IQRs for log2GP37 and log2GP38 were 0.9047 and 0.9858 for the COVID-19 positive group and 0.3248 and 0.4162 for the healthy control group, respectively. Thus, each GP in the healthy control group was distributed more narrowly over a well-contained range, while this was not the case for COVID-19 positive cohort. This observation was further supported by the direct comparison of the HILIC-FLD chromatograms (Fig. 2A), where the N-glycan profiles of blank pooled serum control and healthy control were almost identical (chromatograms 1 and 2). However, the N-GPs of the COVID-19 positive cohort showed considerable variations (selective chromatograms 3, 4, 5, and 6).

### Relative abundance of serum N-glycome changed substantially in the COVID-19 positive cohort

**Fig. 4:**
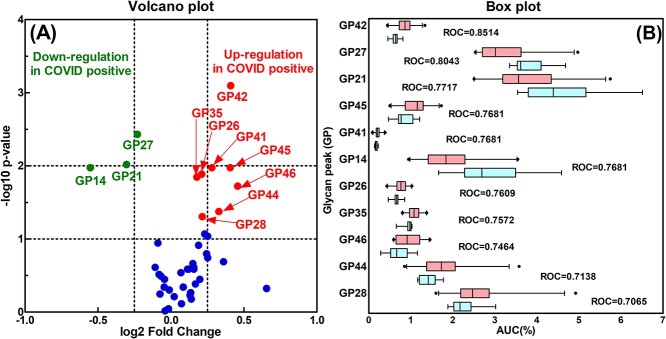
Direct comparison of the percentage or relative abundance level of human serum GPs between COVID-19 positive patients and healthy controls. A) Volcano plot shows the significance of the difference of relative abundance for individual GPs between the 2 cohorts. The *X*-axis represents the log2 transformation of the fold change. The *Y*-axis represents the negative decade logarithm of the significant difference value *P*. Two *P*-value thresholds are indicated (*P* = 0.1 and 0.01). The significant glycan variables above the first threshold were considered as significant changes. Up-regulation is labelled in red, down-regulation is labelled in green, and the rest is labelled in blue. B) Box plot expression of selective human serum GPs down-regulated and up-regulated in COVID-19 positive patients compared to healthy controls. *X*-axis represents the relative abundance of the GP (AUC (%)), and *Y*-axis represents the selected GPs. Each box represents the boundaries of the normal distribution, and the left and right box edge marks the first and third quartile boundaries within each distribution. The bold line within each box indicates the median value of the distribution. Outliers are labelled as black-filled circle. AUC value from ROC curve is displayed to indicate the discriminatory ability of the selected GPs between COVID-19 positive patients and healthy controls.

We further analyzed the composition of the serum N-glycomic profiles and found that the relative abundance (AUC%) of several individual N-GPs was substantially changed in the COVID-19 positive group compared to the healthy control group. To confirm these observations, the relative abundance of the 46 major GPs was analyzed by the Mann–Whitney test and ROC analysis. Figure 4A showed a volcano plot in which GPs 26, 28, 35, 41, 42, 44, 45, and 46 were up-regulated significantly, while GPs 14, 21, and 27 were down-regulated substantially in the COVID-19 positive cohort compared to healthy controls. Figure 4B showed a box plot of the relative abundance of the identified 11 GPs that demonstrated significant difference between the cohort groups. ROC analysis showed the potential in using specific differences in GPs to distinguish COVID-19 positive samples from negative counterparts, including GPs 42 and 27 (AUC for ROC analysis > 0.80, *P* < 0.005) and GPs 21, 45, 41, 14, 26, 35, 46, 44, and 28 (AUC for ROC analysis > 0.70, *P* < 0.05). The complete relative abundance data for all the GPs were shown in Supplementary Information ST IV.

### Specific serum N-glycan subclasses elevated substantially in COVID-19 positive cohort

**Fig. 5:**
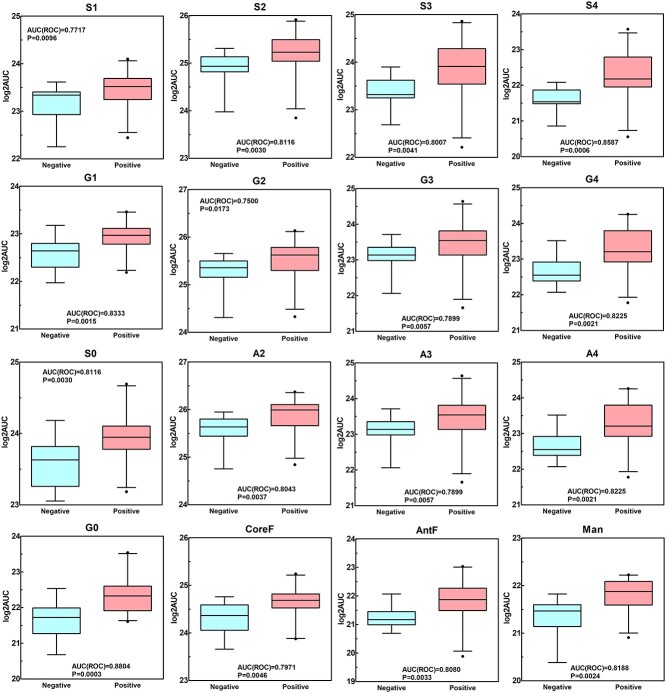
Direct comparison of selective derived traits of glycan subclasses between COVID-19 positive patients and healthy controls. The lower and upper bars connected to each box indicate the boundaries of the normal distribution and the lower and upper box edges mark the first and third quartile boundaries within each distribution. The bold line within the box indicates the median value of the distribution. Outliers are labelled as black filled circle. *Y*-axis represents the log2 transformed AUC for each trait (log2AUC). AUC value from ROC curve and *P* value generated from Mann–Whitney test is displayed to indicate the discriminatory ability of the trait between COVID-19 positive patients and healthy controls. S0: asialylation, S1: monosialylation, S2: disialylation, S3: trisialylation, S4: tetrasialylation, G0: agalactosylation, G1: monogalactosylation, G2: digalactosylation, G3: trigalactosylation, G4: tetragalactosylation, A2: biantennary, A3: triantennary, A4: tetra antennary, CoreF: core fucosylation, AntF: antennary fucosylation, man: mannose.

Apart from individual serum N-GPs, glycan subclasses sharing certain structural features were also compared quantitatively between COVID-19 positive and healthy control groups. The glycan subclasses included sialylation, galactosylation, fucosylation, multiple branched antennae, and high mannose. Each glycan subclass was analyzed by the ROC test and Mann–Whitney test as plotted in Figure 5. The calculation and complete data for the glycan subclasses were shown in Supplementary Information ST III and ST IV. The overall abundance expressed as log2AUC value of each glycan subclass was significantly higher in COVID-19 positive samples compared to healthy controls. Among the glycan subclasses, the log2AUC of total agalactosylation (G0) and tetrasialylation (S4) subclasses for COVID-19 positive differentiated significantly from the healthy control samples (AUC for ROC analysis > 0.85, *P* < 0.001). The abundance for neutral (S0), bisialylated (S2), trisialylated (S3), monogalactosylated (G1), tetragalactosylated (G4), fucosylated (AntF), high mannose (Man), biantennary (A2), and tetraantennary (A4) glycan subclasses also showed significant differentiating ability (AUC for ROC analysis > 0.80, *P* < 0.005). As far as the relative abundance of the glycan subclasses was concerned, only tetrasialylated glycans (S4(%)) demonstrated excellent diagnostic accuracy to distinguish the COVID-19 positive cohort from healthy controls (AUC for ROC analysis = 0.8007, *P* = 0.0041).

### Serum N-glycomic signatures to distinguish COVID-19 positive from healthy cohort

The serum N-glycomic profiles reflect the levels of 23 most abundant glycoproteins in human serum, with immunoglobulins G, A, and M accounting to more than 50% of the abundance (Clerc et al. 2016). Although there were no specific single or multiple GPs exclusively present or absent in COVID-19 positive serum samples, statistical analysis showed several serum N-GPs including GPs 6, 16, 17, 26, 28, and 35, and subclass traits including G0, S3, and S4 (expressed as log2 transformation) significantly up-regulated in the COVID-19 positive cohort compared to the healthy controls (AUC for ROC analysis > 0.80, *P* < 0.005, as shown in Figs 2, 3, 5, and Supplementary Information ST IV). Additionally, the analysis of the relative abundance of the N-GPs and subclass traits (expressed as AUC%) showed 8 GPs were up-regulated and 3 GPs down-regulated. However, there was no significant diagnostic capability to distinguish COVID-19 positive from healthy cohort from the overall analysis of relative abundance of GPs or subclass traits (AUC for ROC analysis < 0.80, *P* > 0.005, as shown in Fig. 4 and Supplementary Information ST IV) except for GP27(%), GP42(%), and S4(%), which individually showed excellent diagnostic performance accuracy (AUC for ROC analysis > 0.80, *P* < 0.005, (Fig. 4 and Supplementary Information ST IV). The variability of the GPs from the COVID-19 positive samples was extensive, whereas the N-glycomic profiles for healthy cohort were well-contained with very narrow IQR.

It has been well-established that choosing appropriate normalization of data is essential for discovery of low abundant glycan biomarkers (Uh et al. 2020). Therefore, with the aim of drawing unbiased logical conclusion, we conducted unsupervised data normalization and statistical analysis without any prior defined variable parameters. Log2 transformation of the AUC can provide relative molar quantification between glycan species since the InstantPC fluorescent dye derivatizes each glycan in a 1:1 molar ratio. While relative abundance expressed as AUC% can provide glycan compositional information. The total serum glycan content was normalized to 100% for each sample (COVID-19 positive or negative).

**Fig. 6:**
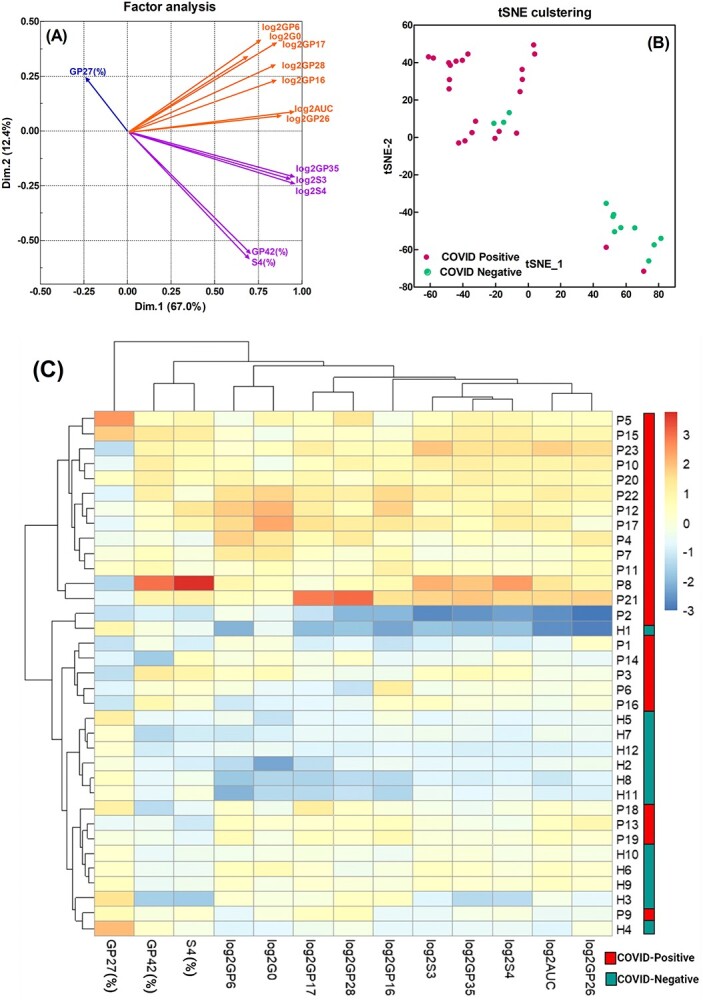
Clustering analysis for potential classification of COVID-19 positive from healthy controls with A) factor analysis, B) tSNE analysis, and C) hierarchical clustering heatmap. A) Factor analysis plot to demonstrate correlation between the significant glycome variables and dimensions. B) tSNE representation with COVID-19 positives and negatives colored as red and green, respectively. C) Hierarchical clustering heat map rows display the 35 investigated serum samples as displayed in SSupplementary Material Table S1 with 23 COVID-19 positive patients (P) and 12 healthy controls (H, 10 individual healthy controls and 2 biological blank pooled serum). Columns indicate the 13 significant serum glycome variables. The dendrogram on the side shows the clustering of COVID-19 positive and controls, and the dendrogram on top shows the clustering of serum glycome variables.

In addition to the above statistical analysis, multivariate factor analysis was performed to confirm the 13 identified serum N-glycome variables to potentially classify COVID-19 positive from healthy controls, including absolute quantification of GPs 6, 16, 17, 26, 28, 35, total AUC, and subclasses G0, S3, and S4 (expressed as log2 transformation), and relative abundance of GPs 27 and 42, and subclass S4 (expressed as AUC%) as shown in Figure 6A. T-distributed stochastic neighbor embedding (tSNE) and hierarchical heatmap clustering analysis were performed to identify N-glycomic variables that distinguished the COVID-19 positive samples from healthy controls. As shown in Figure 6B, tSNE analysis classified the investigated serum samples into 2 major clusters: the positive cluster consisting of 21 COVID-19 positive and 3 healthy controls, the negative cluster consisting of 9 healthy controls and 2 COVID-19 positive. As shown in Figure 6C, the hierarchical heatmap classified the investigated serum samples into 2 major groups: 1 group consisting of 19 COVID-19 positive and 1 healthy control, while the other group consisting of 15 samples with 11 healthy controls and 4 COVID-19 positive.

## Discussion

The COVID-19 pandemic caused by SARS-CoV-2 represents one of the most significant threats to global human health. Currently, COVID-19 is diagnosed routinely by viral ribonucleic acid (RNA) using polymerase chain reaction (PCR)-based techniques or by serological and immunological assays that rely on detection of host antibodies or antigenic proteins in infected individuals following collection of oropharyngeal or nasal mid-turbinate swabs (Carter et al. 2020; Cheng et al. 2020; Udugama et al. 2020; Giri et al. 2021). The science behind the serological and immunological assays is based on the positive association of COVID-19 infection with elevated expression of serum immunoglobins. IgG, IgA, and IgM, against SARS-CoV-2 S1 RBD protein can be detected in human serum within 1–3 weeks after COVID-19 infection. IgG and IgM can arise almost simultaneously. IgM and IgA can decrease rapidly, while IgG can persist for at least several months in majority persons after infection, but the precise duration is unknown (Iyer et al. 2020; Qu et al. 2020; Wolfel et al. 2020; Dan et al. 2021). Therefore, it is reasonable to deduce that the serum N-glycomic alterations may be affected by delayed days of blood drawn after COVID-19 infection due to the changes in these three most abundant antibodies in human serum. Additionally, the clinical severity of COVID-19 demonstrates strong positive correlation with total antibodies titer, independent of age, gender, or comorbidities (Jacofsky et al. 2020; Li et al. 2020; Ma et al. 2020; Marklund et al. 2020; Zhao et al. 2020; Shah et al. 2021). However, the effect of SARS-CoV-2 infection on the overall serum N-glycomic profile has been largely unexplored.

In addition to non-glycosylated albumin, there are at least 23 major glycoproteins detected in human serum potentially involving in multiple biological and pathological processes apart from fibrinogen, which is exclusively present in plasma (Clerc et al. 2016; Merleev et al. 2020). Quantitative glycomic analysis yields significant information about the glycosylation patterns of these major glycoproteins that might have significance to pathology. Therefore, non-supervised mapping of serum N-glycome may hold the potential to identify signatures upon COVID-19 infection and help us better understand this disease, and ideally provide a complementary surveillance strategy allowing timely mitigative interventions. However, serum glycomic studies in disease settings usually suffer from poor analytical resolution, reproducibility, and comparability of results. Here we extended the high-throughput, high-sensitivity 96-well-plate-based glycan profiling workflow described previously (Xie et al. 2021) to identify potential COVID-19-associated serum N-glycomic alterations in a rapid, reliable, and reproducible way.

The serum N-glycome in the COVID-19 positive cohort showed significant up-regulation compared to healthy controls (Fig. 2B–D), with some GPs exhibiting excellent diagnostic performance potential (AUC of ROC > 0.800 as shown in Fig. 3). Additionally, the glycosylation pattern of the COVID-19 positive cohort showed significant glycomic variability as indicated by a wider IQR. However, this was not the case for the healthy cohort, where the glycan patterns from different individuals were well contained within a narrow range, displaying similar profiles if not identical to those obtained from a biological blank of pooled human serum (Fig. 2A and Supplementary Information ST IV). Furthermore, 16 glycan subclass traits were derived from the integrated GPs and found to positively correlate with the COVID-19 positive cohort namely hyperbranching (A4), hypersialylation (S4), hypergalactosylation (G4), and agalactosylation (G0) (Fig. 5 and Supplementary Information ST IV). Thirteen (13) out of a total of 125 serum N-glycome variables (the log2 transformed AUC and relative abundance (%) of the 46 GPs and 16 glycan subclass traits) were identified to distinguish COVID-19 positive from healthy controls (Fig. 6). These included the relative abundance of GPs 27 and 42 and subclass S4 (expressed as AUC%) and absolute quantification of GPs 6, 16, 17, 26, 28, 35, total AUC, and subclasses G0, S3, and S4 (expressed as log2 transformation).

Noticeably, four COVID-19 positive samples (P9, 13, 18, and 19) were grouped together with healthy control (Fig. 6), possibly due to lower concentration of serum immunoglobulins G, A, or M (Table I). The 3 most abundant glycoproteins in human serum (IgG, IgA, and IgM) have characteristic glycan profiles. IgG consists mainly of FA2 (GP5), FA2G1 (GPs 8, 9), FA2BG1 (GP10), FA2G2 (GP14), FA2G2S1 (GP22), IgA consists mainly of A2G2S1 (GPs 19, 20), A2G2S2 (GPs 24, 25) and FA2BG2S2 (GP28), and IgM consists mainly of Man5 (GP6), Man6 (GP11), FA2G2S1 (GP22), and FA2BG2S1 (GP23) (Clerc et al. 2016). Despite the variability in glycan content between COVID-19 positive and negative serum samples, no clear positive linear correlation could be detected in relating this data to the specific immunoglobulin sub-types. However, the use of ELISA to measure specifically immunoglobulins against only SARS-CoV-2 S1 RBD IgG, IgA, or IgM antibody showed distinct differences. It was to be expected that this data correlated with the clinical assignments of the serum samples as positive or negative for COVID-19. The relative content of targeted antibodies between individuals varied and it might be speculated that this related to the timing of sampling or the severity of infection. It may be expected that the measured level of IgM against the SARS-CoV-2 S1 RBD antigen was found to be particularly high as this sub-type is associated with early response to viral infection. However, although the relative content of each antibody against the COVID antigen was determined by reference to individual sub-type standards, their concentrations were not quantified and so any conclusions about the relative content of anti-COVID sub-types would be tentative.

Other publications have shown that IgA levels demonstrate statistically significant correlation with severe and critical status of COVID-19 infection regardless of age, sex, and duration of the symptoms. Measurement of IgA in serum has provided a good diagnostic predictor of outcome in the early stages of infection (Ma et al. 2020; Padoan et al. 2020; Zervou et al. 2021). The above observation is substantiated by our findings with the elevated FA2BG2S2 (GP28), which is exclusively from IgA (Figs 2D, 3, and 4). Of course, one may argue that IgM contains FA2BG2S2 as well (Arnold et al. 2005). However, the overall IgM level itself in human serum is relatively much lower compared to IgG and IgA, in the range of only 0.5–2.0 mg/mL and approximately 5%. Additionally, as far as the N-glycan profiles are concerned, IgM consists mainly of Man5 (GP6), Man6 (GP11), FA2G2S1 (GP22), and FA2BG2S1 (GP23) (Clerc et al. 2016). The combined reasons made the contribution of IgM to serum glycan FA2BG2S2 (GP28) negligible. The observation of FA2BG2S2 (GP28) was increased in COVID-19 patients with high IgM was due to the elevated IgA level in addition to IgM (Fig. 2A and Table I). Additionally, there was a trend observed that increased or decreased level of IgA accompanied by higher or lower AUC value for FA2BG2S2 (GP28) (Table I). However, no clear positive linear correlation between IgA level and FA2BG2S2 (GP28) was found, and the possibly reasons were addressed above.

Our general finding is that the abundance of certain serum glycoproteins comprising specific glycan structures or subclasses are elevated after COVID-19 infection, but to an extent dependent upon the disease severity of different individuals. For example, the N-acetyl methyl groups of the N-acetylglycosamine (GlcNAc) residues located on the bi-, tri-, and tetra-antennary branches of specific serum acute-phase proteins (including mainly alpha-1-acid glycoprotein, haptoglobin, alpha-1-antitrypsin, alpha-1-antichymotrypsin) demonstrates positive correlation with C-reactive protein (Akinkuolie et al. 2014; Otvos et al. 2015). Previous systematic reviews, meta-analysis (Jutzeler et al. 2020; Rodriguez-Morales et al. 2020; Qaisieh et al. 2021; Qi et al. 2021) and machine learning (Kukar et al. 2021) have demonstrated that COVID-19 infected serum show a pronounced increase in CRP level among other blood parameters. This is supported by our findings that CRP associated GPs and subclasses (Burgess and Collaboration CCG 2013) are significantly higher in the COVID-19 positive cohort compared to the healthy control, including GPs16, 41, 42, 44, and 45, and subclasses A2, A4, G4, S3, S4, and AntF.

Previous research has also demonstrated that changes in the level of acute-phase proteins such as alpha-1-acid glycoprotein (AGP) determined by different techniques were identified as biomarkers for the degree and progression of COVID-19 infection (Li and Chen 2020; Lodge et al. 2021). This is also in good agreement with our serum N-glycomic analysis where a significantly increased expression of hyperbranched and hypersialylated GPs and subclasses (GPs 32, 33, 34, 37, 38, 39, 41, 42, 43, 44, 45 and 46, or subclasses S3 and S4) were identified from COVID-19 positive cohort and could be attributed to AGP (Clerc et al. 2016).

Alternatively, higher expression of the glycosyltransferases may be responsible for the enhanced sialylation, fucosylation, and branching (Jeong et al. 2008). Most significantly as terminal components of glycoproteins and glycolipids, the negatively charged sialic acids commonly serve as regulators of molecular and cellular interactions (Kelm and Schauer 1997; Schauer 2009), including virus–sialic acid interactions (Vlasak et al. 1988; Stencel-Baerenwald et al. 2014; Matrosovich et al. 2015; Fung and Liu 2018; Tortorici et al. 2019). Comprehensive glycoproteomic or proteomic experiment by LC–MS after serum proteins enzymatic digestion (Aslam et al. 2017; Shajahan et al. 2017; Suttapitugsakul et al. 2020) can provide valuable and complementary information to support our current glycomic findings, however, it is beyond the scope of the current study. The correlation between glycomics, glycoproteomics, and proteomics will be addressed in a separate article.

Care must be taken to properly interpret the similarity and difference in serum/plasma N-glycome analysis due to the importance of fibrinogen glycosylation. Most proteins present in human plasma or serum are glycoproteins and are similar, except for proteins removed during the coagulation process, including fibrinogen. Fibrinogen is the major protein coagulation factor exclusively present in human plasma with the concentration at 1.5–4.5 mg/mL, yet absent from human serum (Lowe et al. 2004). Regarding its N-glycosylation profiles, fibrinogen predominantly consists of A2G2S1 (GP19) with relative abundance at 53% and A2G2S2 (GP25) with relative abundance at 33% (Adamczyk et al. 2013). Due to the absence of fibrinogen in human serum, A2G2S1 (GP19) is derived mainly from alpha-1B-gycoprotein, haptoglobin, and immunoglobulin A, with the contribution from alpha-2-macrogobulin and apolipoprotein B-100 to a lesser extent (Clerc et al. 2016).

There are of course some limitations to this study in the current form. Since the pandemic began, the COVID-19 infection displays a broad spectrum of symptoms, independent of age, sex, BMI, and ethnic origin. The severity of symptoms and period required for recovery is dependent upon the strain of SARS-CoV-2 infection, varying individual immune, medication treatment, and vaccine status. Therefore, it is impossible to provide a comprehensive multivariate analysis investigation for every aspect of COVID-19 with a limited sample size. The sample size we had access to in this study was relatively small. This included serum samples from 23 confirmed COVID-19 patients, 10 healthy controls, and 2 pooled sera serving as technical blank.

The COVID-19 positive serum samples were from an older cohort (age range 21–92; average age 67) compared with the healthy cohort (age range 18–65; average age 39) as shown in Table I. It is well documented that alterations in serum glycome can be associated with age and gender, and it has been shown that increasing age is associated with a modest decline in overall glycan abundance and IgM level (Merleev et al. 2020). However, this is not the case in the serum N-glycome alterations after COVID-19 infection as found in the current study, where the overall N-glycan abundance (expressed as log2AUC) for the 45 major GPs increased in COVID-19 positive cohort compared to healthy control (except for GP 14).

It is recognized that long-term and large population screening could improve the analytical accuracy of the current analysis presented in our study. However, larger sample sizes and multiple time-based patient serum samples were not available to us. Nevertheless, we determined that the current serum sample set was adequate in identifying any major changes in the overall glycomic profile of serum proteins following COVID-19 infection. Despite these limitations, to the best of our knowledge this is the first report relating human serum N-glycomic profiles to untargeted identification of signatures for COVID-19 in a high-throughput, high-sensitivity manner. By associating serum N-glycomic features with the clinical outcome of COVID-19, this study lays the foundation for future glycomic studies to determine the value of monitoring serum N-glycomic profiles as a surveillance tool for COVID-19, including serum N-glycomic alterations in correlation with the risk of severe symptoms, drug efficacy, different vaccinations, time-course vaccination, or even discovery of N-glycan-related biomarkers for COVID-19 diagnosis. This could be especially beneficial for those recovered from infection with negative PCR or serological lateral flow testing results but still experiencing sustained long-term COVID-19 related consequence (termed as “Long-COVID”). Long-COVID or the post-COVID-19 condition has gradually attracted extensive attention (Wise 2020; Akbarialiabad et al. 2021; Alwan 2021; Alwan and Johnson 2021; Beasley et al. 2021), comprehensive characterization of post-acute sequelae of COVID-19 are still to be comprehensively described (Al-Aly et al. 2021). This untargeted serum N-glycomic profiling described here may serve as one of several techniques for long-COVID surveillance to help a better understand of this disease and consequently to improve patients’ diagnosis.

## Materials and methods

### Materials and reagents

The AdvanceBio Gly-X N-Glycan Prep with InstantPC kit, 96-ct (Cat NO: GX96-IPC) consisting of three modules, including Gly-X deglycosylation module (Cat NO: GX96-100), Gly-X InstantPC labelling module (Cat NO: GX96-101), and Gly-X InstantPC cleanup module (Cat NO: GX96-102), was donated by Agilent Technologies (Santa Clara, California, USA). HPLC-grade acetonitrile was purchased from Sigma Aldrich (St. Louis, Missouri, USA) and Milli-Q water was used in all preparations. All the common chemicals were purchased from Sigma Aldrich (St. Louis, Missouri, USA).

### Study designs

The objective of this study was to identify potential human serum N-glycosylation alterations upon SARS-CoV-2 infection, and gain better understanding of COVID-19 disease. The serum sample set containing 20 COVID-19 positive samples with varying IgG, IgM, and IgA antibody levels and 10 healthy control samples (Cat NO: CoV-PosSet-S1) and COVID-19 positive sample with high IgG content (Cat NO: CoV-PosG-S-100), high IgM content (Cat NO: CoV-PosM-S-100), and high IgA content (Cat NO: CoV-PosA-S-100) were purchased from RayBiotech (Peachtree Corners, Georgia, USA). The COVID-19 status was confirmed with reverse transcription polymerase chain reaction (RT-PCR), antigen, and/or antibody serology tests. Healthy control serum samples from a pool of different donors (Cat NOs: H4522 and S1-M) used as technical quality controls or blanks were purchased from Sigma-Aldrich (St. Louis, Missouri, USA). Research was performed in accordance with relevant guidelines and regulations. The representative and complete information for the COVID-19 and healthy control serum samples is included in Table I and Supplementary Information Excel worksheet ST I, respectively.

### ELISA determination of antibodies to SARS-CoV-2 S1 receptor binding domain protein

The in vitro indirect enzyme-linked immunosorbent assay (Ollis et al. 2015) kits for detection of SARS-CoV-2 S1 RBD protein human IgG (Cat NO: IEQ-CoVS1RBD-IgG), IgM (Cat NO: IEQ-CoVS1RBD-IgM), and IgA (Cat NO: IEQ-CoVS1RBD-IgA) were purchased from RayBiotech (Peachtree Corners, Georgia, USA). Quantitative measurement of human IgG, IgM, and IgA antibody against the SARS-CoV-2 S1 RBD protein in human serum was carried out according to the manufacturer’s instruction and as referenced previously (Adler et al. 1981; Amanat et al. 2020; Gong et al. 2021; Luo et al. 2022). Briefly, human serum samples (1 μL) were diluted 1,500 times for IgG measurement, and 500 times for IgM and IgA measurement by adding 1,499 and 499 μL of 1× sample diluent, respectively. Additionally, dilution series (1,000, 333.3, 111.1, 37.04, 12.35, 4.12, and 1.37 unit/mL) of COVID-19 positive control samples from inactivated serum containing SARS-CoV-2 S1 RBD protein human IgG, IgM, and IgA antibodies were prepared by 1× sample diluent. The 1× sample diluent served as the blank. Samples and prepared positive controls (100 μL) were added to appropriate wells of SARS-CoV-2 S1 RBD protein coated 96 well microplates, as well as the additional albumin protein coated 96 well microplates in the cases of IgM and IgA measurement. The plates were incubated for 1 h at room temperature (21 °C) with gentle shaking. The solution was discarded, and each well was washed 4 times with 300 μL of 1× wash buffer. Biotinylated anti-human IgG, IgM, and IgA antibody solution in 1× assay diluent (100 μL) was added to each well, and the plates were incubated for 30 min at room temperature with gentle shaking. The solution was discarded, and each well was washed 4 times with 300 μL of 1× wash buffer. Horseradish peroxidase (HRP)–streptavidin solution (100 μL) was added to each well, and the plates were incubated for 30 min at room temperature with gentle shaking. The solution was discarded, and each well was washed 4 times with 300 μL of 1× wash buffer. The 3, 3, 5, 5′-tetramethylbenzidine (TMB) 1-step substrate reagent (100 μL) was added to each well, and the plates were incubated for 15 min at room temperature in the dark with gentle shaking. Stop solution (50 μL) was added to each well, and the absorbance at 450 nm was read immediately. The sample solution was diluted by adding a suitable amount of stop solution if the absorbance was out of detection range. The mean absorbance at 450 nm for each set of duplicate samples was calculated following subtraction of the blank reading. The values (unit/ml) were determined from calibration curves established on a log–log scale with standard positive controls for IgG, IgM, and IgA provided by RayBiotech (Peachtree Corners, Georgia, USA).

### Preparation of InstantPC labelled glycans

The preparation of InstantPC labelled glycans from human serum was carried out according to the manufacturer’s instruction and described in detail previously (Xie et al. 2021). Briefly, human serum (1 μL) was diluted with 19 μL of 4-(2-hydroxyethyl)-1-piperazineethanesulfonic acid (HEPES) buffer (50 mM, pH 8.0) to make a final volume of 20 μL. Gly-X denaturant (2 μL) was added to the 20 μL of serum solution, mixed thoroughly and incubated at 90 °C for 3 min. After leaving at room temperature for 2 min, 2 μL of N-Glycanase working solution was added, mixed thoroughly, and incubated at 50 °C for 5 min. InstantPC dye solution was prepared by dissolving one vial of InstantPC dye with 150 μL of the accompanying solvent and mixed well. The InstantPC dye solution (5 μL) was added to the above prepared human serum sample, and incubated at 50 °C for 1 min. The Load/Wash solution (150 μL of 2.5% formic acid/97.5% acetonitrile) was added to each sample, and then the entire sample (179 μL) was transferred to each well of the Gly-X Clean-up plate containing 400 μL of the load/wash solution. After passing the solution through the clean-up plate by applying a vacuum, samples were washed with 600 μL of the load/wash solution 3 times. InstantPC labelled glycans were eluted with 100 μL of Gly-X InstantPC eluent (160 mM ammonium formate/10% (v/v) acetonitrile, pH 4.4). The collected InstantPC labelled glycan solutions were analyzed immediately without further treatment, or alternatively stored at −20 °C for future analysis.

### InstantPC labelled glycan profiling by HILIC-FLD

The profiles of InstantPC labelled glycans from human serum were determined by HILIC-FLD using Acquity I class UPLC equipped with Acquity UPLC Glycan BEH Amide Column (130 Å, 1.7 μm, 2.1 × 150 mm, SKU: 186004742) under the control of Empower software (Waters Corporation, Milford, Massachusetts, USA). Similar separation performance for the InstantPC labelled glycans was achieved on the 1290 Infinity II ultra-high performance liquid chromatography system (UHPLC) equipped with AdvanceBio Glycan Mapping column (Rapid resolution HD, 300 Å, 1.8 μm, 2.1 × 150 mm, Part No: 859700-913) under the control of OpenLab software (Agilent Technologies, Santa Clara, California, USA). Each system consists of a binary solvent pump, autosampler, and a fluorescence detector. The detector for InstantPC was set with excitation and emission wavelengths at 285 and 345 nm, respectively. The InstantPC labelled glycans from human serum were injected at a volume of 1 μL without any prior treatment. The InstantPC glycans were separated with 50 mM ammonium formate (pH 4.4) as solvent A and acetonitrile as solvent B. After initial system equilibrium for 1.5 min with 27% of 50 mM ammonium formate (pH 4.4) and 73% acetonitrile (v/v) at a flow rate of 0.5 mL/min, the separation was carried out by a linear gradient of 73–62% of acetonitrile (v/v) at a flow rate of 0.5 mL/min in 40 min, followed by a linear gradient of 62–53% of acetonitrile (v/v) at a flow rate of 0.5 mL/min in 12 min. After washing the system under 30% of acetonitrile (v/v) at a flow rate of 0.4 mL/min for 3 min, complete system equilibrium under 27% of 50 mM ammonium formate (pH 4.4) and 73% of acetonitrile (v/v) at a flow rate of 0.5 mL/min for another 15 min was carried out to ensure good chromatographic reproducibility. Samples were maintained at 5 °C before injection and the separation column temperature was 60 °C. The systems were routinely calibrated using AdvanceBio InstantPC Maltodextrin ladder (Cat NO: GKPC-503) donated by Agilent Technologies (Santa Clara, California, USA). The correlation between glucose unit (GU) value and chromatographic retention time T (min) was fitted to 5th order polynomial function to obtain the standard curve.

### Batch correction and data preprocessing

The chromatographic GPs from the HILIC-FLD analysis were processed with the built-in software for automated peak picking and integration. Individual GPs were analyzed on the basis of the correlation between measured retention time and GU values generated from the 5th order polynomial standard calibration curve against AdvanceBio InstantPC Maltodextrin ladder under identical conditions. The chromatograms were all separated in the same manner into 46 major GP and the glycan structures were assigned as described previously (Saldova et al. 2014; Haakensen et al. 2016) and independently confirmed in the lab by hydrophilic interaction ultra-performance liquid chromatography coupled with electrospray ionization mass spectrometry (HILIC–UPLC–ESI–MS) and exoglycosidase sequential digestion, and the complete assignment for InstantPC labelled human serum N-glycans is shown in Supplementary Information Excel spreadsheet ST II. The glycan structures were represented by following the Symbol Nomenclature for Glycans (SNFG) system (Varki et al. 2015). In addition to the 46 directly measured GPs, 16 derived glycan subclass traits were calculated as described previously with minor modifications (Saldova et al. 2014; Pavic et al. 2018) and the calculation formula was shown in Supplementary Information Excel spreadsheet ST III. These derived glycan subclass traits averaged specific glycosylation features (sialylation, galactosylation, fucosylation, mannosylation, and branching and extension) across different individual glycan structures.

To remove experimental variation from measurements, batch correction and normalization were performed on glycan data. The AUC for each individual GP, glycan subclass trait, and total GPs was subjected to further log2 transformation before analysis. Additionally, total area normalization was applied, where the percentage (or relative abundance) of each GP and subclass trait were calculated by the integrated peak AUC of each GP divided by AUC from total GPs of corresponding chromatogram, which represented the composition of glycans and subclasses in a serum sample. Both the log2 transformed absolute quantity data and compositional data were used for further statistical analysis.

### Statistical analysis

Logistic regression model was carried out to identify potential alterations in serum glycome to distinguish COVID-19 positive patients from healthy controls (Nick and Campbell 2007; Stoltzfus 2011; Sperandei 2014). It generates the coefficients of the formula to predict a logit transformation of the probability of presence of the characteristic of interest, $\mathrm{logit}(P)=\ln (\mathrm{odds})=\ln\big (\frac{P}{1-P}\big)$, where *p* represents the probability of presence of characteristics, and 1 − *P* represents the probability of absence of characteristics. Additionally, Mann–Whitney nonparametric test was used for comparison between the 2 cohorts by using the following formula: $U=\min ({U}_1,{U}_2)=\min \big({n}_1{n}_2+\frac{n_1({n}_1+1)}{2}-{R}_1,{n}_1{n}_2+\frac{n_2({n}_2+1)}{2}-{R}_2\big)$, where *n*_1_ and *n*_2_ represent the size, and *R*_1_ and *R*_2_ represent the adjusted rank-sum for sample 1 and 2, respectively (Sheskin, 2022). The diagnostic potential of significantly differed individual GPs and subclasses was further analyzed by receiver operator characteristic (Hosmer and Lemeshow 2000; Zou et al. 2007; Mandrekar 2010; Hajian-Tilaki 2013; Behnke et al. 2021). The ROC curve was created by plotting the true positive rate $\big(\mathrm{TPR}=\frac{\mathrm{TP}}{P}=\frac{\mathrm{TP}}{\mathrm{TP}+\mathrm{FN}}\big)$ against the false positive rate $\big(\mathrm{FPR}=\frac{\mathrm{FP}}{N}=\frac{\mathrm{FP}}{\mathrm{FP}+\mathrm{TN}}\big)$ at various threshold settings, where *P* is the number of real positive cases in the data, TP is true positive, FN is false negative, *N* is the number of real negative cases in the data, FP is false positive, and TN is true negative. The AUC value generated from ROC test provides an aggregate measure of performance across all possible classification thresholds and is an effective way for overall summary of diagnostic accuracy. An AUC of 0.7–0.8 is considered acceptable, 0.8–0.9 is considered excellent, and more than 0.9 is considered outstanding, while 0.5 suggests no discrimination at all. The complete statistical analysis result data for GPs and subclasses are shown in Supplementary Information Excel spreadsheet ST IV. To identify potential relationships to distinguish COVID-19 positive patients from healthy controls, logistic regression model, principal component analysis, hierarchical clustering heatmap, and t-SNE under R environment (version 4.1.1) (Rizzo, 2019), a free software environment for statistical computing and graphics, and packages of blorr, FactoMineR, factoextra, pheatmap, Rtsne, ggplot2 were used.

## Supplementary Material

Serum_glycome_for_COVID-19_surveillance_supplementary_data_cwac051
